# Pulverization of Waste Polyvinyl Chloride (PVC) Film by Low Temperature Heat Treatment and Properties of Pulverized Product for Blast Furnace Injection as Alternative Fuel

**DOI:** 10.3390/polym14091689

**Published:** 2022-04-21

**Authors:** Guang Wang, Sixian Liu, Hongqiang Zhang, Jingsong Wang, Qingguo Xue

**Affiliations:** State Key Laboratory of Advanced Metallurgy, University of Science and Technology Beijing, Beijing 100083, China; liusixian2022@126.com (S.L.); wangjingsong@ustb.edu.cn (J.W.); xueqingguo@ustb.edu.cn (Q.X.)

**Keywords:** waste polyvinyl chloride (PVC), pulverization, heat treatment, de-chlorination, blast furnace injection

## Abstract

Recycling of waste plastics is of great significance for human society. The pulverization of waste film plastics is a key technical link in the development of collaborative utilization of waste plastics in the steel industry. In this study, waste polyvinyl chloride film plastics were first heated at different temperatures; then the de-chlorination ratio pulverization and the properties of the pulverized products closely related to blast furnace injection, such as powdery properties, combustion and explosiveness, were further analyzed. The weight loss ratio increased significantly with an increase in temperature and was not obvious between 370 °C and 400 °C. The highest de-chlorination ratio was approximately 84% at 370 °C, and the relative chlorine content in the product was 9%. The crushing performance of heat-treated polyvinyl chloride film increased with increasing temperature. Before 370 °C, there were more pores in the samples, and the surface of the sample seemed to be damaged with the temperature was further increased. The pulverized polyvinyl chloride had better fluidity and strong jet flow compared to industrial injection coals. At the same time, compared with other carbonaceous materials, it also exhibited better combustion performances. The pulverized polyvinyl chloride belonged to non explosiveness substance despite its high volatile content. The obtained results demonstrated that the pulverized polyvinyl chloride obtained under the present conditions could be used for blast furnace injection to some extent.

## 1. Introduction

Polyvinyl chloride (PVC) is the second-most produced thermoplastic by volume, after polyethylene. It has the characteristics of easy processing, wear resistance, acid and alkali resistance, flame retardancy, and excellent electrical insulation. Therefore, it is widely used in pipes, window framing, floor coverings, roofing sheets, and cables [1,2]. Due to the strong demand for PVC, China’s PVC production capacity has maintained an annual growth ratio of 20% since 2000. Notably, it is expected that by the end of 2050, the cumulative PVC waste in the environment will exceed 600 million tons in China [3,4].

In recent years, the question of the disposal of PVC waste has gained increasing importance in the public discussion. At present, there are four commonly used PVC waste processing technologies: mechanical recycling, landfilling, incineration, and chemical recycling [5]. The mechanical recycling method involves directly using PVC waste plastics after simple pretreatment (such as collection, sorting, washing, and grinding of the material) or mixing them with other polymers to produce blends. This method is simple and feasible, but requires high quality waste plastics [6]. Landfill treatment is common, but landfilled waste plastics can cause serious problems, such as land occupation, soil structure damage, environmental pollution, and the loss of chemical calorific value of waste plastics [7]. Incineration of waste PVC produces a large amount of HCl, which can damage the incineration equipment, and increase the investment and operation costs of the incineration and disposal process. In addition, PVC waste incineration will inevitably produce dioxins and other toxic gases, which will result in environmental pollution [8]. Chemical recycling is the conversion of PVC back into shorter chains for reuse in petrochemical or polymerization processes following cracking, gasification, hydrogenation, or pyrolysis [2]. Compared with other waste plastic processing technologies, this type of recycling has high potential for heterogeneous and contaminated plastic waste material, where separation is either not economically viable or not completely technically feasible [6].

The fossil fuel energy-intensive blast furnace ironmaking process of steel industry operates at high temperature and under high reduction potential with the function of energy conversion, which can provide an easier path for the collaborative utilization of waste plastics in large quantity and low cost. Up to the present, the application of waste plastics in the iron and steel industries has been studied extensively. In 1996, the steel plant in Germany achieved blast furnace injection of waste plastics for the first time in the world [9]. After sorting and removing harmful impurities, the waste plastics were pulverized into plastic particles (smaller than 10 mm) and injected into the blast furnace having an injection capacity of 70,000 tons per year. Japan first implemented blast furnace injection of waste plastics in the Keihin plant. In this method, the chlorine-containing plastics were removed in advance and the rest was crushed and granulated (the maximum particle size was approximately 6 mm) for being injected into the blast furnace together with hot air. The experimental injection amount was as high as 200 kg/tHM [10].

Whether waste plastics are used for blast furnace injection or gasification reactions to produce gas fuel, the particle size of waste plastics is very important. Particle size affects the reaction rate and conversion efficiency by affecting the mass and heat transfer between the particles [11]. Different processes have different requirements of the particle size of the raw material. The blast furnace requires that 80% of the total mass of injection coal should a particle size smaller than 0.074 mm. However, a smaller particle size increases the production cost and technical difficulty. Asanuma et al. [12] pulverized a mixture of various plastics into 0.2–0.4 mm through heat treatment, however, they didn’t pay attention to the behavior of PVC plastics. Wang et al. [13] obtained low chlorine hydrochar from PVC by hydrothermal carbonization; however, the hydrothermal treatment equipment of PVC is significantly eroded by the formed HCl, as it is in the solution state. 

To the best of our knowledge, few studies have reported the dry pulverization of waste PVC film and the application characteristics of the pulverized product in the blast furnace ironmaking process. In the present research, waste PVC film plastics were pulverized by heat treatment at different temperatures without using water, and the de-chlorination ratio and properties of the pulverized products closely related to blast furnace injection as solid fuel were further analyzed. The process not only help efficiently use the PVC waste plastics resources in the municipal solid waste, but also avoids bringing a large number of harmful substances into the next process.

## 2. Materials and Methods

### 2.1. Raw Materials

The PVC film plastic used in this experiment was a decoration material, also known as PVC foam board, widely used in model making and advertising. The thickness was 0.1 cm, and the chemical composition is shown in Table 1. Before the experiment, the PVC film was cut into pieces of about 2.0 cm × 2.0 cm for subsequent use. The coke, graphite, and anthracite were crushed into the size smaller than 180 μm and dried at 100 °C for 10 h for subsequent use.

### 2.2. Experimental Methods

First, the PVC film pieces were subjected to low temperature heat treatment in N_2_ atmosphere; then, the cooled heat-treated products were ground and sieved. The macroscopic morphology, microstructure, de-chlorination ratio, and properties for blast furnace injection of the pulverized heat treatment products were studied. 

#### 2.2.1. Low Temperature heat Treatment Experiment

**Figure 1:**
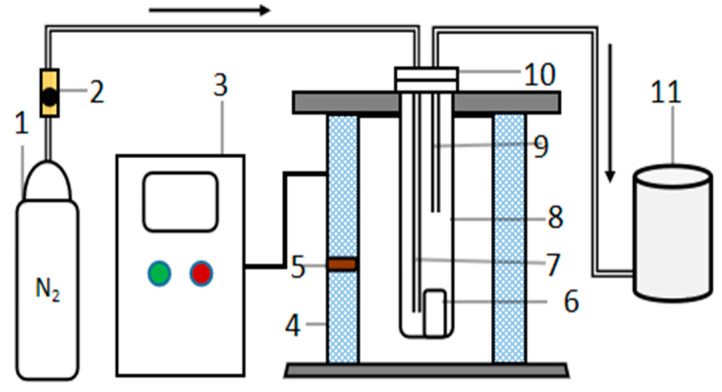
Schematic diagram of the experimental setup. 1: gas cylinder, 2: flowmeter, 3: control cabinet, 4: furnace body, 5: heating element; 6: crucible; 7: gas inlet, 8: furnace tube, 9: gas outlet, 10: flange, 11: tail gas washing tank.

A total of 10 g of PVC film were placed in stainless steel crucibles. The crucible was a cylinder having a diameter of 5 cm and height of 9 cm. The heating equipment was a tubular shaft furnace, and its heating element were U-shaped MoSi_2_ rods, which were arranged around the furnace. During the test, high purity N_2_ flow at 3 L/min was introduced from the bottom of the furnace tube to prevent oxidation in the furnace. The gas outlet from the top of the furnace tube was connected to the tail gas filtering device to completely absorb the HCl from the tail gas. The schematic of the setup is shown in Figure 1. Before the experiments, the furnace temperature was set to a fixed temperature required for each run (280, 310, 340, 370, 400, 430, and 460 °C). When the furnace reached the desired temperature, the stainless steel crucible containing the PVC film pieces was placed into the furnace and heated for 30 min.

#### 2.2.2. Pulverization and Screening

After heat treatment, the PVC film samples were cooled to room temperature in an N_2_ atmosphere. The weight of the PVC film products was recorded before pulverization to calculate the weight loss ratio. The crusher crushed the sample continuously with a rotating speed of 20,000 rpm. The heat treatment products obtained at each temperature were crushed for 20 s, and the crushed samples were screened using sieves of different sizes (12, 16, 28, 45, 60, 80, and 150 mesh).

#### 2.2.3. Microstructure Characterization

The microstructure of the PVC film heated at different temperatures was compared using scanning electron microscope (SEM) images, and the distribution and content of selected elements in the products were analyzed using an electron probe microanalyzer (EPMA). The samples for microstructure characterization were cut and drowned in the resin. The cross section was further polished and sprayed a carbon film before the characterization test. 

#### 2.2.4. Thermogravimetric Analysis

The combustion experiment of the carbonaceous raw materials was performed by thermogravimetric analysis. The equipment used was an thermal analyzer (SDT Q600, TA Instruments, New Castle, DE, USA). Approximately 10 mg samples were taken and heated from room temperature to 1000–1250 °C at 10 °C/min in air, with a gas flow rate of 100 mL/min. The combustion performance of pulverized PVC, coke, graphite, and anthracite were analyzed.

#### 2.2.5. Powdery Property Analysis

The fluidity and jet flow properties of carbonaceous raw materials were tested by a multifunctional powder physical property tester (MT-02, SEISHIN, Japan). The particle sizes of raw materials were smaller than 180 μm. The fluidity and jet flow of each sample were measured three times, and their average values were recorded. 

### 2.3. Evaluation Indicator

#### 2.3.1. De-Chlorination Ratio of Polyvinyl Chloride (PVC) Film (α)

To evaluate the de-chlorination of PVC films after heat treatment at different temperatures, the chlorine content of PVC raw materials and products after heat treatment was determined by ion chromatography (IC) designated ion test. The de-chlorination ratio at different heat treatment temperatures was calculated, using the expression given below:

α = (M_0_ − M_T_)/M_0_ × 100% (1)

where M_0_ was the chlorine amount in the PVC film (%), and M_T_ was the chlorine amount in the heated PVC film at a certain temperature.

#### 2.3.2. Crushing Performance Index (φ)

To compare the crushing performance of PVC heat treatment products under different conditions. and considering that the product having particle size less than 0.18 mm is easy to be used for thermochemical reaction, the proportion of crushing products having a particle size less than 0.18 mm in the total material mass is defined as the crushing performance index, and its expression is given below:

φ = W_0.18_/W (2)

where W_0.18_ was the mass having particle size smaller than 0.18 mm in the heat treatment product (g), and W was the total mass of the heat treatment product (g).

#### 2.3.3. Combustion Performance Index (S_N_)

To compare the combustion performance of pulverized PVC heat treatment product and various carbonaceous materials, the combustion performance index was introduced to characterize the combustion performance of each carbonaceous material, and its value was calculated using the following formula [14]:

S_N_ = (DTG_max_ × DTG_mean_)/(T_i_^2^ × T_f_) (3)

where S_N_ was the combustion performance index, min^−2^·°C^−3^; DTG_max_ was the maximum weight loss rate (%/min); DTG_mean_ was the mean weight loss rate (%/min); T_i_ was the ignition temperature (°C); and T_f_ was the burnout temperature (°C). Notably, the greater the S_N_ value, the better the combustion performance of the fuel, and vice versa.

## 3. Results and Discussion

### 3.1. Low Temperature Heat Treatment Experiment of Polyvinyl Chloride Film

**Figure 2:**
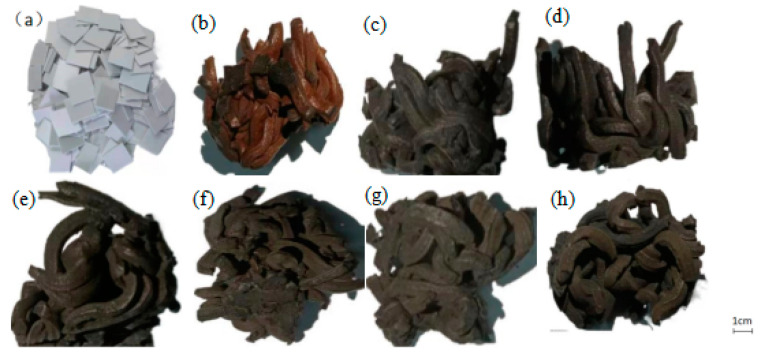
Morphology of PVC film heated at different temperatures: (**a**) PVC film pieces, (**b**) 280 °C, (**c**) 310 °C, (**d**) 340 °C, (**e**) 370 °C, (**f**) 400 °C, (**g**) 430 °C, (**h**) 460 °C.

The PVC film was subjected to constant temperature heat treatment at 280, 310, 340, 370, 400, 430, and 460 °C for 30 min. The morphology of the PVC obtained at different temperatures is shown in Figure 2. It can be seen that the PVC film shrank from film pieces to strips and the formed strips closely gathered together. At 280 °C, the PVC film in the center was light yellow, indicating that the temperature was not high enough. The PVC film was not carbonized. With the increase in temperature, the light-yellow part of the heat treatment products gradually disappeared, and the volume reduction was more.

**Figure 3:**
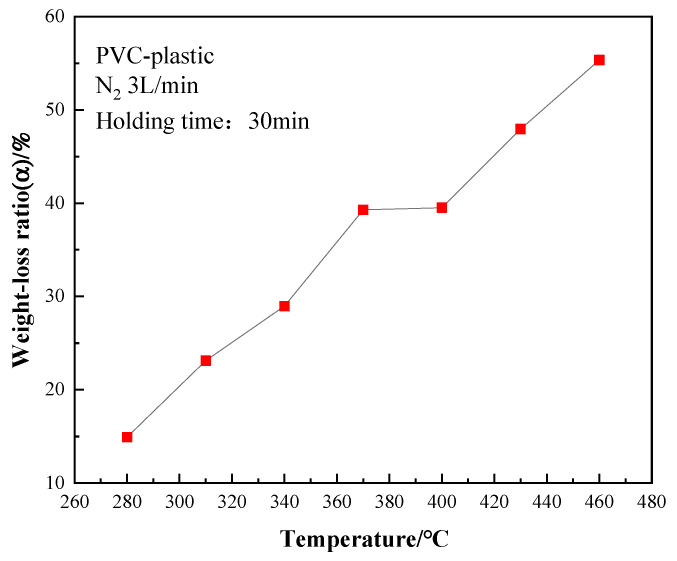
Relationship between heat treatment temperature and weight loss ratio of PVC film.

The relationship between the heat treatment temperature and the weight loss ratio of the PVC samples is shown in Figure 3. It can be seen that the weight loss ratio of PVC film was very low at 280 °C. With the increase of temperature, the weight loss ratio continued to increase, and a flat region began to appear at 370 °C. After 400 °C, the weight loss ratio increased immediately with increasing temperature. The two weight loss stages (<370 and >400 °C) of the PVC films were also accompanied by different reactions. In the first stage, the main reaction was the removal of HCl from the PVC film. With increasing temperature, the internal energy of PVC film increased, the fracture of the C-Cl bond intensified, and the amount of removed HCl increased. It was also accompanied by the volatilization of small molecular substances during thermal degradation; thus, the weight loss ratio increased significantly [15]. After 400 °C, the weight of the PVC film entered the second loss stage, the cracking of the main chain (crosslinked polyene) in the PVC film intensified, many small molecular compounds were produced, and the weight loss ratio increased sharply. At 370–400 °C, the change in the weight loss ratio was very small, which meant no decomposition reaction occurred. 

**Figure 4:**
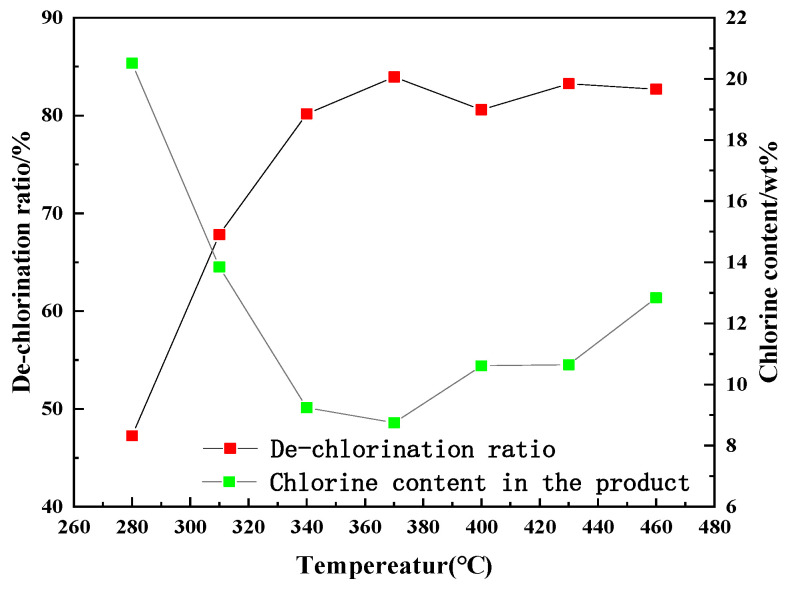
Chlorine content and de-chlorination ratio of PVC film at different temperatures.

Figure 4 shows the de-chlorination ratio of the PVC film and the chlorine content in the product at different heat treatment temperatures. It can be seen from the figure that at a temperature lower than 370 °C, the de-chlorination ratio increased significantly with the increasing temperature. When the temperature was 370 °C, the de-chlorination ratio reached a maximum value of 84%. After 370 °C, the de-chlorination ratio decreased, portraying a stable trend at approximately 80%. The C-Cl bonds in the structure of PVC have a relatively lower binding energy than the C-C and C-H bonds. Therefore, the C-Cl bond in the PVC broke first with the increasing temperature. Moreover, the HCl released from PVC can catalyze the de-chlorination reaction; therefore, the ratios of de-chlorination portrayed an increasing trend [16]. After 370 °C, hydrocarbons were mainly produced, and the de-chlorination ratios decreased slightly and then showed a stable trend [17]. Notably, the chlorine content in the product was negatively correlated with the de-chlorination ratio. The chlorine content obviously decreased before 370 ℃ and gradually increased after 370 °C. At 370 °C, the chlorine content of the product was approximately 9%. Some chlorine should have been trapped in the residue as a result of interaction with the additives in the PVC film sample [18]. It can be concluded that the most suitable heat treatment temperature for the de-chlorination of PVC film in the present experimental condition was approximately 370 °C.

### 3.2. Pulverization of Polyvinyl Chloride after Heat Treatment

The pulverization experiment was performed to crush and screen the heat treatment products of the PVC film. The influence of temperature on the crushing performance index of the products was determined. The optimal heat treatment conditions of PVC film could be obtained by considering the aforementioned de-chlorination results at the same time.

**Figure 5:**
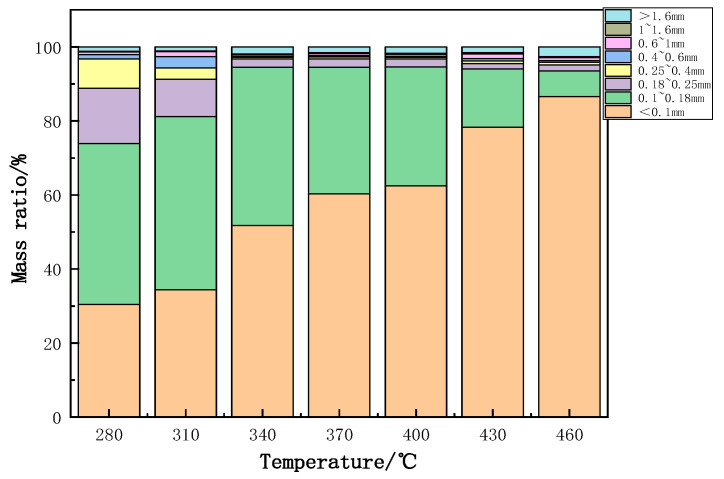
Particle size distribution of crushed PVC film heated at different temperatures.

**Figure 6:**
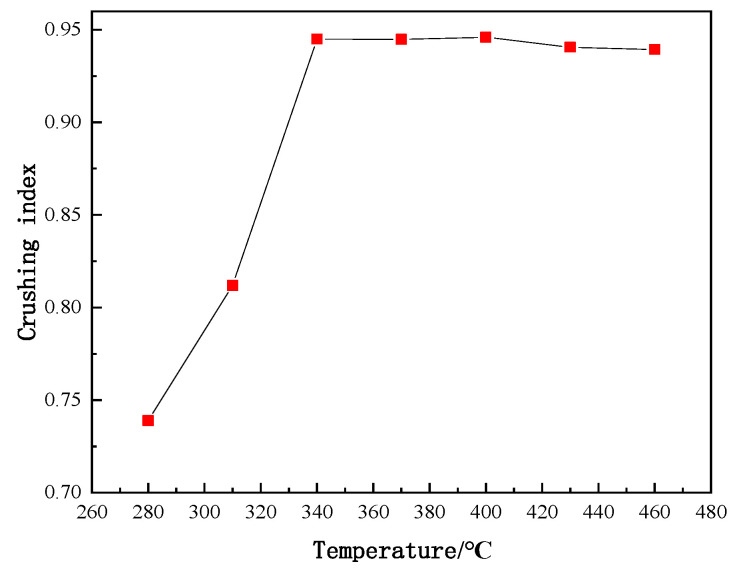
Crushing performance index of PVC film heated at different temperatures.

After being heated at various temperatures, the PVC film was crushed and sieved into different particle sizes. As shown in Figure 5, with an increase in temperature, the proportion of powder having a small particle size, such as 0.10 mm, gradually increased. At the temperature higher than 340 °C, the proportion of powder with the size larger than 0.25 mm became very little, which was around 2.50 to 3.00%. In the present work, the mass proportion of powder having particle size less than 0.18 mm was defined as the crushing performance index to roughly illustrate the particle size of the crushed products in total. The variation of the crushing performance index with temperature is shown in Figure 6. Notably, the crushing performance index was small at lower temperatures, such as 0.3 at 280 °C. Before 340 °C, the temperature had a significant influence on the crushing performance index. With increasing temperature, the crushing performance index increased rapidly due to obviously improved embrittlement by pyrolysis compared to the initial polymer state. The crushing performance index (>0.9) tended to be stable after 340 °C. Because the de-chlorination ratio of the PVC film reached its peak at 370 °C, this temperature of 370 °C was considered to be ideal for obtaining the optimal crushing performance of the PVC film. For the convenience of the following description, PVC heat treatment products at 370 °C were denoted as PVC370. Furthermore, the proximate analysis of newly obtained PVC370 was performed in dry basis and the content of fixed carbon, volatile matter, and ash of PVC370 were 19.56%, 54.75%, and 25.69%, respectively.

### 3.3. Microstructure Analysis of Polyvinyl Chloride after Heat Treatment

**Figure 7:**
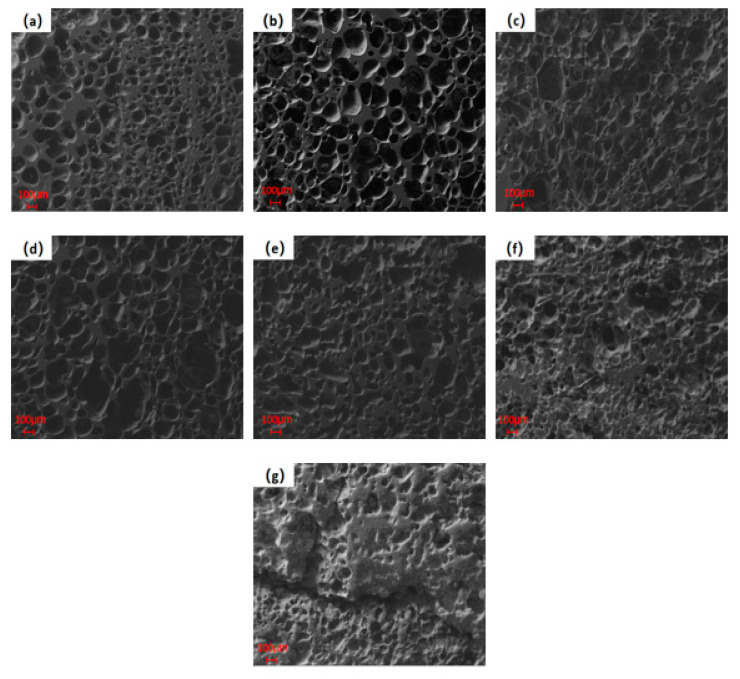
Scanning electron microscope (SEM) images of heated of PVC film at different temperatures: (**a**) 280 °C; (**b**) 310 °C; (**c**) 340 °C; (**d**) 370 °C; (**e**) 400 °C; (**f**) 430 °C; (**g**) 460 °C.

SEM images of the PVC film heated at various temperatures are shown in Figure 7. Many unevenly sized pores could be observed at 280 °C. The width of the large pores was approximately 200 μm, and the width of the small pores was around 30 μm. This indicated that only a part of the volatiles in the PVC film was released at 280 °C and it was mainly the lateral chain in the PVC macromolecules being broken [19]. As the temperature increased, the pore size of the samples became larger, which indicated that the amount of volatiles that diffusing outward increased [20]. Before 370 °C, there were more pores in the samples, and they were deeper. At 370 °C, the samples had much larger pores, with the size of approximately 400 μm. When the temperature was further increased, the surface of the sample seemed to be damaged. Many small pores were connected together and became shallower, and some disappeared at 430 °C. Notably, at 460 °C, the cracks began to appear, and the carbon matrix structure was much denser. Only a small part of the main chain in the PVC film broke before 370 °C. After 400 °C, a large number of carbon frameworks were thermally cracked into smaller molecular substances and volatilized, which resulted in the denser residual carbon matrix [21].

**Figure 8:**
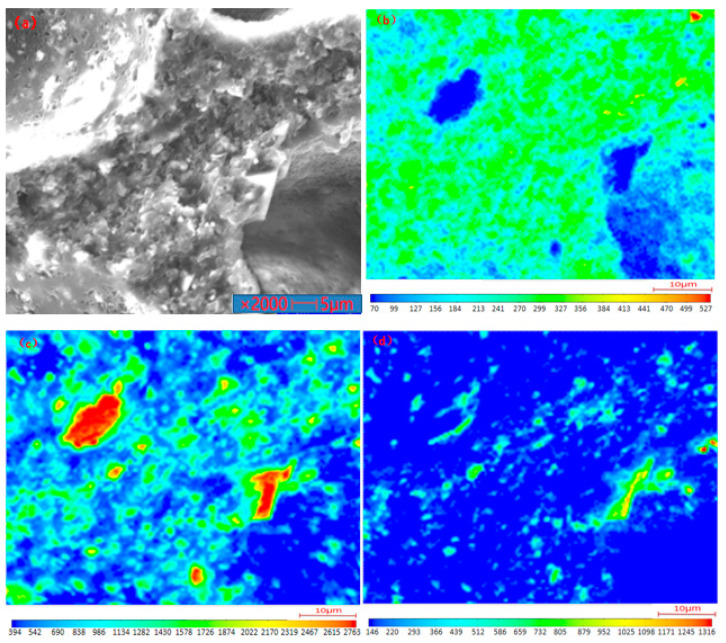
Mapping analysis of heated PVC film (370) by EPMA: (**a**) original image; (**b**) Cl element distribution; (**c**) Ca element distribution; (**d**) O element distribution.

The PVC film sample heated at 370 °C was analyzed using EPMA to investigate the existing state and content of chlorine in the product. Figure 8 shows the distribution of Ca, Cl, and O. Cl was evenly distributed in the matrix around the holes on the sample surface (as shown in Figure 8b). Notably, Cl was widely distributed in the carbon matrix, indicating that residual chlorine was trapped in the residual matrix, which was difficult to remove. The Ca content in the sample was distributed in small pieces and concentrated at the edge of the hole (as shown in Figure 8c). Generally, some additives are added into the pure raw polyvinyl chloride material during the production of commercial polyvinyl chloride. However, the PVC manufacturers are reluctant to tell the exact chemical composition of the additives. In the present work, the used PVC film sample contained a little CaCO_3_ additive. Cl also appeared where the Ca was distributed, but not in the place where Ca was most concentrated.

**Figure 9:**
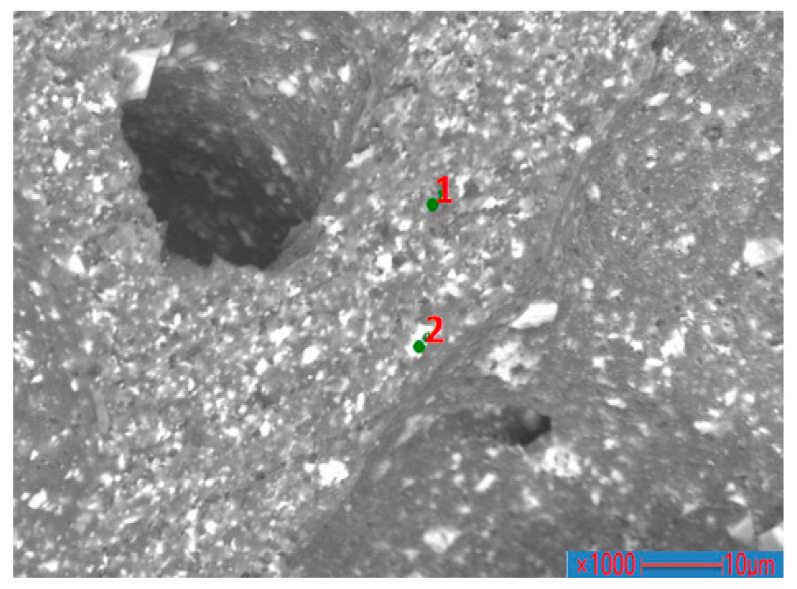
Micro zone analysis of heated PVC film (370 °C) by EPMA: (**1**) point 1; (**2**) point 2.

Micro zone analysis was further conducted on the sample, as shown in Figure 9. The corresponding element content was obtained at the selected position on the sample, as listed in Table 2. It can be seen that the carbon matrix of the sample contained a large amount of C element and less O, Cl, and Ca elements. There were many Ca and O elements in the white zone, such as point 2, indicating that the white zone was mainly a compound of Ca. By comparing the amounts of Cl and Ca elements of point 2, it could be concluded that part of the Ca existed in the form of compounds combined with Cl, and the remaining Ca existed in the form of CaCO_3_ or CaO. 

### 3.4. Analysis of Pulverized Polyvinyl Chloride for Blast Furnace Injection

The injection of pulverized coal into a blast furnace can replace coke, reduce the cost of pig iron, and enrich reducing gas. It has become the prevailing technology in the blast furnace ironmaking process [22]. In the present study, the pulverized polyvinyl chloride after heat treatment was proposed to replace the coal or coke. With the help of the new technology, the consumption of fossil fuel can be reduced and the waste plastics will also be recovered. Therefore, it is meaningful to systematically study the powdery, combustion, and explosive characteristics of the pulverized polyvinyl chloride.

(1) Analysis of powdery properties of pulverized polyvinyl chloride

In a system containing powder, the particles of the powder are interrelated, and some special flow characteristics appear when the particles rub against each other. Therefore, the study of fluidity and jet flow is of great significance for the processing, transportation, storage, and packaging of powder. Studying these parameters can also provide practical reference for the metallurgical, chemical, and other industries, which consume powdery raw materials. The fluidity and jet flow of pulverized polyvinyl chloride and two injection coals were measured. The fluidity performance of the powder included four factors: natural slope angle, compression ratio, scoop angle, and uniformity. The compression ratio was obtained by the loose density and tap density, and the other factors were directly measured by the test. Additionally, the jet flow characteristics, including the crash angle, angle of difference, dispersity, and fluidity, were also studied. The crash angle, angle of difference, and dispersity were obtained directly from the test. Based on the principle of the Carr index [23], the properties of pulverized polyvinyl chloride (i.e., PVC370) and two injection coals in transportation, storage, and production were evaluated, and the results were listed in Table 3. As seen in the table, PVC370 showed better fluidity performance than the two kinds of injection coals. The natural slope angle of PVC370 was the smallest, indicating that the relative friction between the materials of this sample was small, which was conducive to improve the flow performance. The jet flow performance of PVC370 was weaker than those of the injection coals, but it still reached the strong jet flow degree. This indicated that the powdery properties of pulverized PVC product after heat treatment could meet the injection requirements of blast furnace ironmaking operation to partially replace coal or coke.

(2) Combustion properties of pulverized polyvinyl chloride

**Figure 10:**
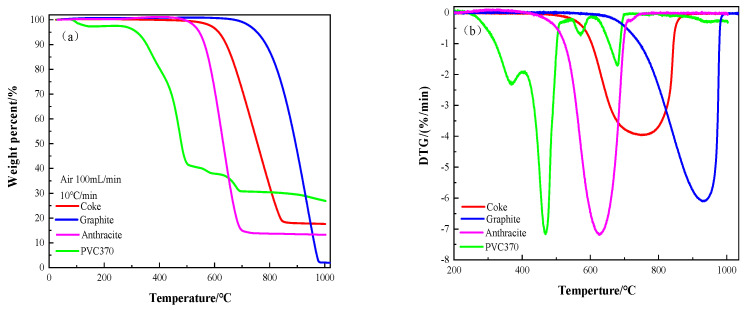
Combustion curves of each carbonaceous material: (**a**) weight loss curves, (**b**) weight loss rate curves.

The weight loss curves of PVC370 and other carbonaceous materials during combustion are shown in Figure 10. The corresponding reaction performance parameters were obtained according to the weight loss curve, as shown in Table 4. It can be seen from the figure that coke, graphite, and anthracite lost the weight in only one stage. Graphite almost lost all the weight at the temperature of 997 °C. The weight loss ratio of coke was approximately 83% at the temperature of 880 °C. The weight loss ratio of anthracite was approximately 87% at the temperature of 749 °C. Compared with other carbonaceous materials, PVC370 was more prone to react. The weight loss curve of PVC370 can be divided into three stages: (1) 264–508 °C (weight loss ratio 42.58%); (2) 508–586 °C (weight loss ratio 3.36%); and (3) 586–703 °C (weight loss ratio 7.20%). The weight loss of the first stage consisted of the devolatilization and combustion of small molecules, and the removal of HCl, and the second stage mainly consisted of the combustion of the residual carbon backbone. The combustion of PVC370 was almost completed in the third stage; therefore, the rate of weight loss decreased [24]. The order of initial combustion temperatures increased as follows: PVC370 (264 °C) < anthracite (446 °C) < coke (533 °C) < graphite (660 °C). The maximum reaction rate of PVC370 was similar to that of anthracite and higher than those of graphite and coke. The combustion performance index (S_n_) of each carbon material was calculated using Equation (3). The combustion performance index of PVC370 was significantly higher than those of the other carbon materials. Coke and graphite had almost the same combustion indexes, and both were much smaller than those of the other two carbonaceous materials. The combustion characteristic indexes decreased in the sequence of PVC370 > anthracite > coke > graphite.

(3) Explosive property of pulverized polyvinyl chloride

In the blast furnace ironmaking process, the injection coal is ground into the grain size 80% less than 0.074 mm to improve the combustion rate. However, the pulverized coal after fine grinding has a large specific surface area, which is prone to explosion during or after the grinding. Therefore, blast furnace injection is very concerned with the explosiveness of coal, especially for highly volatile bituminous coal [25]. The explosiveness of coal is in direct proportion to the level of volatile matter. The higher the volatile content, the stronger the explosiveness of pulverized coal [26]. The volatile content of PVC heat treatment products is higher than that of bituminous coal, so it is necessary to study its explosiveness.

Generally, the explosiveness of pulverized coal is reflected by the length of flame. The flame length of pulverized coal is measured by long tube explosion performance tester. In practical engineering applications, it is generally believed that if the length of the return flame formed by the detonation of the measured pulverized coal is greater than 600 mm, the coal can be classified as highly explosive. If it is between 400~600 mm, the coal has medium-intensity explosiveness, and if it is less than 400 mm, the coal has weak explosiveness. If there are only rare sparks or no sparks at the fire source, it exhibits non-explosiveness [27,28]. The return flame length of coal injected into the blast furnace is between 20~50 mm, which can realize safe injection [26]. The test results of explosiveness of PVC370, anthracite, mixed coal, and bituminous coal are shown in Table 5. It can be seen that return flame length of PVC370 and anthracite was almost zero and was much smaller than traditional bituminous coal although the volatile content of PVC370 was much higher. This was a very interesting experimental result. It might be related to the flame retardants that added into PVC during its production to prevent fire for safety [29]. Furthermore, the ignition point of PVC370 was 326 °C, which was lower than 398 °C for anthracite and 334 °C for mixed coal. The combustion characteristics of PVC370 were better than anthracite, and PVC370 was non-explosive. Therefore, PVC370 was more suitable for blast furnace injection than anthracite if the chlorine amount injected into blast furnace together with pulverized PVC was controlled to meet the requirements of total chlorine load of blast furnace ironnaking operation. 

## 4. Conclusions

(1) The PVC film samples slowly shrank in volume with increasing temperature from 280 °C to 460 °C. The de-chlorination ratio increased significantly with increasing temperature before 370 °C. The de-chlorination rate was up to 84% at 370 °C, and the chlorine content in the product was 9%. The de-chlorination ratio decreased slightly as the temperature continued to rise, then showed no change. The pulverization performance increased with the temperature. Overall, the optimum heat treatment temperature of the PVC film was 370 °C.
(2) Pores would form during the heat treatment of PVC film samples due to the emission of volatile. Before 370 °C, there were more pores in the samples, and they were deeper. At 370 °C, the samples had much larger pores, with the size of approximately 400 μm. When the temperature was further increased, the surface of the sample seemed to be damaged. The microstructure of the PVC heat treatment product obtained above 400 °C was denser. Some of the Cl remained in the residual matrix or combined with Ca, and it was difficult to remove this part of Cl.
(3) After heat treatment at 370 °C (i.e., PVC370), the fluidity of pulverized polyvinyl chloride was better than the two kinds of injection coals. The jet flow was weaker than that of the injection coals, but it still reached a strong degree. PVC370 had a lower initial combustion temperature and higher combustion rate than other carbonaceous materials. PVC370 was classified as a non-explosive substance despite its high volatility.
(4) The low temperature heat treatment of PVC can help remove chlorine and improve the pulverization performance of thermoplastic PVC film. The pulverized PVC heat treatment product can meet the relevant requirements of blast furnace injection to replace coal or coke from the powdery, combustion, and explosive points of view based on present study.

## Figures and Tables

**Table 1:** Proximate and elemental analysis of raw materials.

Material	Proximate Analysis (wt %)			Elemental Analysis (wt %)					
	V_d_	A_d_	FC_d_	C	H	O	N	S	Cl
PVC film	70.10	16.57	13.33	33.67	2.68	17.19	<0.3	0.61	33.09
Coke	1.64	12.16	86.20	83.81	1.66	3.85	0.62	0.50	-
Graphite	-	-	100	-	-	-	-	-	-
Anthracite	6.4	11.1	81.4	77.71	1.21	8.19	0.55	1.12	-

Note: V_d_ was the volatile in dry basis, A_d_, was the ash in dry basis, FC_d_ was fixed carbon in dry basis.

**Table 2:** Chemical composition of selected points (at%).

Point No.	C	O	Cl	Ca
1	87.68	6.69	2.76	2.87
2	55.52	23.37	2.93	17.88

**Table 3:** Powdery properties of pulverized PVC370 and injection coal.

Physical Property	Pulverized PVC Heat Treatment Product	Injection Coal 1	Injection Coal 2
Tap-density (g/mL)	0.611	0.89	0.801
Compression ratio (%)	24	21	33
Natural slope angle (°)	22.3	37	26.2
Crash angle (°)	17.6	19	11
Angle of difference (°)	4.7	18	15.2
Scoop angle (°)	22.8	43	15.5
Dispersity (%)	26.9	25	33.9
Uniformity (D_60_/D_10_)	6.55	5.94	7.86
Fluidity index	87	75	76
Degree of fluidity	Good	Good	Good
Jet flow index	68	82	81
Degree of jet flow	Strong	Very strong	Very strong

**Table 4:** Combustion characteristic parameters of different samples.

Materials	Peak	T_i_ (°C)	T_f_ (°C)	T_m_ (°C)	DTG_max_ (%/min)	S_N_ × 10^9^	t_g_ (min)
PVC370	1	264	513	467	7.16	456.58	24.78
	2	542	606	572	0.71	1.56	6.38
	3	606	703	680	1.60	4.46	9.78
Anthracite	1	446	749	628	7.14	136.10	30.43
Graphite	1	660	997	934	6.12	40.87	33.83
Coke	1	523	880	760	3.95	37.25	35.84

Note: T_m_ was the temperature corresponding to the peak of the weight loss rate, t_g_ was the reaction time.

**Table 5:** Results of explosiveness of carbonaceous materials.

Materials	T/°C	L/mm	V_d_/%
PVC370	326	0	54.75
Injection anthracite	398	0	7.4
Injection mixed coal	334	30	18.18
Bituminous coal [30]	293	>700	33.25

Note: T was the ignition point temperature, L was the explosion flame length.

## Data Availability

The data presented in this study are available on request.
